# Sphingosine-1-phosphate stimulates colorectal cancer tumor microenvironment angiogenesis and induces macrophage polarization via macrophage migration inhibitory factor

**DOI:** 10.3389/fimmu.2025.1564213

**Published:** 2025-06-16

**Authors:** Fang Wu, Zhaode Feng, Xuan Wang, Yingcong Guo, Bingcong Wu, Shuheng Bai, Ning Lan, Min Chen, Juan Ren

**Affiliations:** ^1^ Department of Radiotherapy, The First Affiliated Hospital of Xi’an Jiaotong University, Xi’an, China; ^2^ Center for Mitochondrial Biology and Medicine, The Key Laboratory of Biomedical Information Engineering of Ministry of Education, School of Life Science and Technology, Xi’an Jiaotong University, Xi’an, Shaanxi, China; ^3^ Department of Kidney Transplantation, Nephropathy Hospital, The First Affiliated Hospital of Xi’an Jiaotong University, Xi’an, Shaanxi, China; ^4^ College of Life Sciences, Inner Mongolia University, Hohhot, China

**Keywords:** sphingosine-1-phosphate, tumor-associated macrophages polarization, angiogenesis, colorectal cancer, scRNA-seq

## Abstract

**Background:**

Colorectal cancer (CRC) is the most common gastrointestinal malignancy with extensive reprogramming of sphingolipid metabolism. However, the role and mechanisms of sphingosine-1-phosphate (S1P), a key bioactive molecule in sphingolipid metabolism, remain insufficiently characterized. Therefore, this study integrated multi-omics data to elucidate the characteristics and functions of S1P within the tumor microenvironment (TME) and investigated its role in angiogenesis through *in vitro* experiments.

**Methods:**

We used bulk RNA sequencing data sets (RNA-seq) to study the prognostic value and clinicopathological characteristics of the increased synthesis of S1P. In order to elucidate the contribution of S1P to the complexity of the tumor microenvironment, we employed intercellular communication analysis and functional enrichment analysis at the single-cell transcriptome (scRNA-seq) level. The expression of Sphingosine kinase 1 (SPHK1) in human tissues was verified by immunohistochemical staining (IHC). Then, we inhibited the synthesis of S1P by suppressing SPHK1 at the cellular level to explore the changes in the pro-angiogenic function of tumor cells and M2-like macrophages, as well as the direction of macrophage polarization.

**Results:**

S1P activity is elevated in the TME of CRC, and the increased synthesis of S1P suggests poor prognosis and early metastasis. intercellular communication analysis indicates that high S1P epithelial cells can promote angiogenesis and influence the polarization of tumor-associated macrophages (TAMs) through the macrophage migration inhibitory factor (MIF) pathway. TAMs were grouped according to gene expression patterns, in which, PCLAF+ cluster TAMs showed significantly high S1P activity, contributing to tumor growth and angiogenesis. IHC demonstrated elevated levels of SPHK1 protein expression in CRC tumor tissues. Inhibition of the synthesis of S1P in tumor cells and macrophages suppressed macrophage M2 polarization levels and reversed the pro-angiogenic phenotype by inhibiting VEGFA protein expression. Spatial transcriptomics revealed a correlation between the distribution of SPHK1 and M2-like macrophage.

**Conclusions:**

By integrating multi-omics data and further cellular experiments, we propose that inhibition of S1P may play an important role in inhibiting angiogenesis and reversing M2-type macrophage polarization, demonstrating its anti-tumor efficacy in CRC.

## 1 Introduction

Colorectal cancer (CRC) is one of the most common gastrointestinal malignancies worldwide (1). Among adults under 50, CRC has become the leading cause of cancer-related deaths in men and the second leading cause in women (2). Since 1990, the incidence of CRC in adults under 55 years of age has rising by 1%–2% annually (1–4). Despite advances in treatment of CRC, ensuring long-term survival for younger patients remains a significant challenge. Angiogenesis, a critical process in CRC metastasis, is a key therapeutic target. In advanced unresectable CRC, chemotherapy combined with anti-angiogenic therapy remains the standard of care (3, 5–7). However, drug resistance has become a roadblock for long-term survival of colorectal cancer patients. Lipid metabolism reprogramming within the tumor microenvironment (TME) plays a pivotal role in CRC progression, including tumor proliferation, migration, and resistance to anti-angiogenic therapy (8, 9). Thus, understanding the metabolic characteristics of lipids in the TME is essential for identifying novel therapeutic targets to overcome drug resistance and improve patient outcomes.

Sphingolipids, essential components of cellular membranes, are not only involved in maintaining membrane fluidity but also serve as signaling molecules that regulate tumor development, invasion, and metastasis (10–13). Among these, sphingosine-1-phosphate (S1P) and ceramide are two key bioactive lipids. Sphingolipid metabolism is an extremely complex biological process. Ceramide is the central hub of this process (14). S1P is synthesized by the phosphorylation of sphingosine via sphingosine kinase 1/2 (SPHK1/2), whereas sphingosine is derived from the hydrolysis of ceramide by ceramide- hydrolase (15). In CRC, the imbalance between S1P and ceramide is considered to be one of the most important causes of recurrent metastasis (13). S1P, known for its anti-apoptotic and pro-proliferative effects, plays a central role in CRC progression and the development of chemoresistance. Transporter proteins that export S1P to the extracellular space are central to the emergence of chemoresistance in CRC (15). The S1P signaling pathway normally functions through the S1P receptor (16), but can also work through non-S1P receptor-dependent pathways. In other malignancies, such as breast cancer, S1P facilitates the interactions between cancer cells and other cells within TME by promoting tumor-derived cytokine production, which drives angiogenesis and lymph-angiogenesis (17, 18). Besides, it has been demonstrated that endothelial cells expressing the Spns2(S1P transporter protein) are involved in maintaining S1P gradients within lymphatic tissues, thereby promoting cancer cell metastasis by inhibiting the activity of cytotoxic T cells (19). However, systematic single-cell-level studies are lacking to fully elucidate the metabolic characteristics and intercellular interaction mechanisms of S1P within the TME of CRC.

SPHK1 is a key enzyme responsible for S1P synthesis in the cytoplasm, with its expression closely correlated to S1P levels (20). PF-543(https://www.medchemexpress.cn/PF-543.html) has been reported to reversibly bind to SPHK1, thereby inhibiting its enzymatic activity and reducing intracellular S1P levels. SPHK1 exhibits distinct expression patterns across various tumor types, and in most cases, its abnormal overexpression is associated with poor prognosis (21). Previous studies have demonstrated that the SPHK1/S1P axis has been shown to promote metastasis through the regulation of apoptosis and autophagy in CRC (21, 22). Studies have shown that SPHK1/S1P/S1PR1 axis may be a key pathway in the development of chronic colitis-related tumors (23). However, few studies systematically explored the precise mechanisms through which SPHK1/S1P mediates intercellular interactions and modulates immune cell functions within the TME.

Tumor-associated macrophages (TAMs), an important component of the TME, can differentiate into two distinct phenotypes, M1-like and M2-like, in response to various stimuli within the TME (24, 25). M1-like macrophages exhibit a strong pro-inflammatory phenotype, which activates immune responses and eliminates tumor cells. The tumor microenvironment predominantly favors the polarization of macrophages toward the M2-like phenotype (26). M2-like macrophages suppress immune responses and play an important role in promoting epithelial-mesenchymal transition (EMT), angiogenesis, and immunosuppression (27). It has been suggested that in non-small cell lung cancer, dysregulation of sphingolipid metabolism may enhance tumor angiogenesis by promoting macrophage polarization toward the M2-like phenotype (28). However, the relationship between S1P activity and TAMs polarization in the TME of CRC remains unclear.

**Figure 1:**
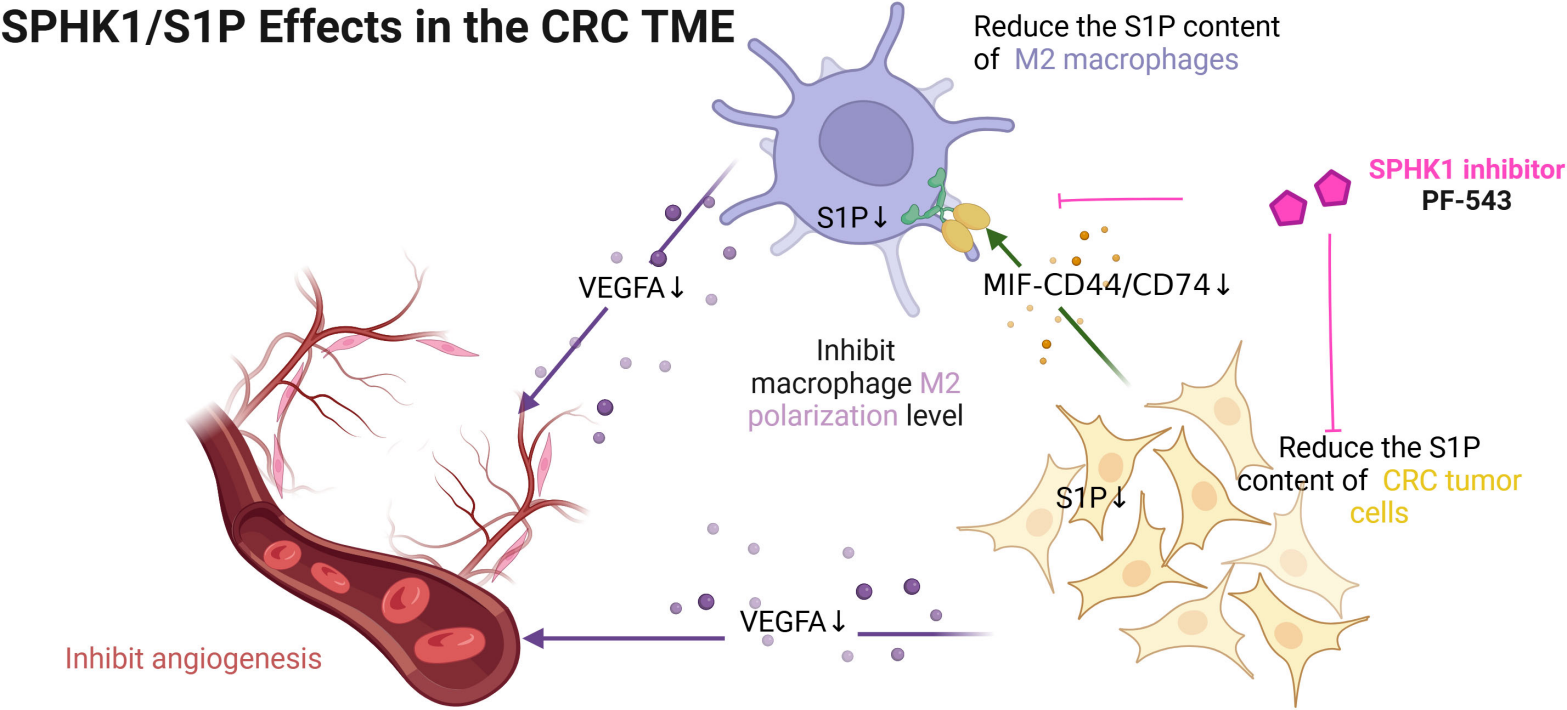
The mechanism of this study. [Figure created with BioRender.com].

By integrating bulk and single-cell transcriptomic data, our study demonstrates that elevated SPHK1/S1P activity in CRC cells is associated with increased pro-angiogenic function, earlier metastasis, and poorer clinical outcomes. Experimental validation revealed that targeting SPHK1/S1P signaling suppresses VEGFA expression in tumor cells and M2-like macrophages, thereby reducing angiogenesis and inhibiting tumor progression. Furthermore, downregulation of S1P levels disrupts epithelial-macrophage communication via the MIF-CD74/CD44 axis limiting M2 macrophage polarization. These findings reveal the SPHK1/S1P axis as a potential therapeutic target in CRC. The proposed mechanism is illustrated in **Figure 1**.

## 2 Materials and methods

### 2.1 Raw data acquisition

Bulk RNA-seq data and clinical information of TCGA-COAD and TCGA-READ were downloaded from the UCSC Xena (https://xenabrowser.net/datapages/), containing 51 normal tissue samples and 638 CRC samples. Samples with incomplete survival and clinical information were excluded to obtain a training set of 606 CRC patients for this study. scRNA-seq datasets were downloaded from the GEO (https://www.ncbi.nlm.nih.gov/) database. Among them, we collected 13 pairs of CRC tumor tissues and adjacent normal tissues from 12 patients from the GSE166555 dataset (29), and obtained 34 tumor tissue samples from the others (GSE144735, GSE132257, GSE132465) (30). Spatial transcriptome data for a sample of primary CRC tumor tissue was downloaded from 10X Genomics Software (https://www.10xgenomics.com/) (31). The key enzymes in the process of sphingolipid metabolism pathway were obtained from KEGG database (https://www.kegg.jp) (32). Detailed information on the datasets can be found in **Supplementary Table S1**.

### 2.2 Processing of scRNA-seq data

The raw gene expression matrices from all samples were aggregated and converted into Seurat objects using the “Seurat” R package (v4.0) (33). Quality control criteria excluded cells with >6,000 or <200 detected genes, or >5% mitochondrial gene content. Doublets were identified and removed using the “DoubletFinder” R package. Genes expressed in at least three cells were retained for analysis. Cell cycle scoring was performed for the S and G2/M phases using the predefined “cc.genes.updated.2019” gene set in Seurat, and the resulting scores were regressed out following data normalization. Highly variable genes (n = 2,000) were identified using the FindVariableFeatures function. Principal component analysis (PCA) was then performed on these genes to reduce dimensionality. Based on the elbow plot, the top 20 principal components (PCs) were selected for downstream analysis using the “FindNeighbors” and “RunUMAP” functions. Batch effects were corrected using the Harmony algorithm (34). Cell clustering was performed using the “FindClusters” function, exploring resolution values between 0.1 and 1.6, with 0.5 chosen as the optimal setting. Cell clusters were annotated based on cell markers identified in previous literature and the CellMarker2.0 database (http://xteam.xbio.top/CellMarker/) (35–37).

### 2.3 Gene sets acquisition and scoring

For obtaining the gene sets related to tumor sphingolipid metabolism, searching the keyword “sphingolipid metabolism” on the Molecular Signatures Database (MSigDB) website (https://www.gsea-msigdb.org/gsea/msigdb) (38, 39) and selecting those relative to tumor. Eight gene sets reflecting tumor sphingolipid metabolism activity were selected. These gene sets were scored and visualized using the integrated scoring algorithm implemented in the “irGSEA” R package (40). Details of eight gene sets are listed in **Supplementary Table S2**.

A S1P Score was assigned to each colorectal cancer patient by calculating the log2(TPM+1) values difference between the enzyme that synthesizes S1P and that of the enzyme that degrades S1P in the bulk RNA sequence data. Colorectal cancer patients were divided into high_S1P and low_S1P groups based on the median value of the S1P_score.

Differential expression analysis between the high_S1P and low_S1P groups was conducted using the limma package in R, resulting in 70 differentially expressed genes (DEGs). To further refine the S1P-related gene signature, we retrieved 199 genes associated with S1P activity (relevance score > 2) from the GeneCards database (https://www.genecards.org/). The intersection of the 70 DEGs and the 199 S1P-related genes, we obtained a total of 15 genes.

These 15 genes were then used to compute the single-cell S1P activity score (scS1P_score) using the “AddModuleScore” function in the Seurat R package. Each cell was assigned an scS1P_score. Among epithelial cells, we further stratified them into high- and low-S1P epithelial subgroups based on the median value of scS1P_score (**Supplementary Figure S4**). DEGs are listed in **Supplementary Table S2**.

To obtain gene sets related to tumor functions, keywords such as “angiogenesis” and other tumor-related terms were searched on the MSigDB website. The identified gene sets were scored and visualized using the AUCell algorithm. Details of the gene sets are provided in **Supplementary Table S6**.

The normality of data distribution was assessed using the Shapiro-Wilk test, and the homogeneity of variances was evaluated with Levene’s test. Statistical comparisons were performed using the t-test method.

### 2.4 Survival analysis and analysis of correlation

Survival analysis between the high-S1P and low-S1P groups was conducted using the R packages “survival” and “survminer”. The overall survival and expression levels of SPHK1 and VEGFA were analyzed using data from The Cancer Genome Atlas (TCGA) COAD and READ cohorts, accessed via GEPIA2.0 (http://gepia2.cancer-pku.cn/).

Correlation analysis between two genes was performed using the “ggcorplot” R package, which was also utilized to generate correlation visualization plots. Pearson’s correlation coefficient was used to assess associations between normally distributed variables.

### 2.5 Gene set variation analysis and analysis of immune infiltration

Gene sets related to tumor-associated biological functions were retrieved from MSigDB using the R package “gsva” (41). Subsets of epithelial cells and macrophages were scored using the GSVA algorithm, and the results were visualized with the “ggplot2” and “pheatmap” R packages. At the bulk RNA-seq level, immune cell infiltration in high-S1P and low-S1P groups was assessed using the “MCPcounter” algorithm implemented in the “IOBR” R package (42).

### 2.6 Cellchat analysis and molecular docking

To characterize the number and intensity of cell communication in the tumor microenvironment of CRC, the “CellChat” (V2.0) R package was used. “CellChat” objects were created, and the ligand−receptor interaction database was initialized. Expression data were preprocessed for communication analysis, and the “trimean” function was utilized to calculate communication probabilities and infer cell-cell communication networks. Systematic analyses were conducted to identify the roles of MIF signaling in various cell groups (e.g., transmitter, receiver) and to determine the primary contributing signals. The results were visualized using the built-in visualization functions of the “CellChat” package.

Molecular structure predictions and docking site identification for MIF-CD74/CD44 interactions were performed using AlphaFold3 (https://alphafoldserver.com/). The final docking sites were visualized using PyMOL software (version 4.6.0).

### 2.7 Cell culture and conditioned medium collection

The human CRC cell lines SW480, Caco2, HCT116 and the human acute myeloid leukemia (AML) cell line U937 used in this experiment were purchased from the Shanghai Cell Bank of the Chinese Academy of Sciences (CAS, Shanghai, China). Human umbilical vein endothelial cells (HUVECs) were obtained from ScienCell Research Laboratories (Carlsbad, CA, USA). SW480, Caco2 and U937 cells were cultured in Roswell Park Memorial Institute (RPMI)-1640 Medium (Gibco, CA, USA) supplemented with 10% fetal bovine serum (FBS; Gibco, CA, USA) and 1% penicillin and streptomycin as a nourishing medium. HCT116 and HUVECs were cultured in Dulbecco’s Modified Eagle Medium (DMEM; Gibco, CA, USA) with 10% FBS and 1% penicillin-streptomycin. ALL cell cultures were cultured in a 5% CO2 incubator at 37°C.

Caco2 + inhibitor conditioned medium (Caco2 + inhibitor CM): PF-543 was added to the culture medium of Caco-2 cells at a final concentration of 20 ng/mL for 24 hours. These cells were then seeded into six-well plates with fresh culture medium to eliminate any residual PF-543. Upon reaching 70-80% confluency, the cell supernatant was centrifuged and stored at −80°C for subsequent experiments.

Caco2 conditioned medium (Caco2 CM): The same amount of DMSO (the solvent for PF-543) was added to culture medium of Caco-2 cells for 24 hours. These cells were then seeded into six-well plates with fresh culture medium. the cell supernatant was centrifuged and stored at −80°C for subsequent experiments.

Phorbol 12-myristate 13-acetate (PMA; MCE, USA) was added to the culture medium of U937 cells at a final concentration of 100 ng/ml. After 24 hours of stimulation, macrophage adherence was observed. The tumor cell-derived conditioned medium was then replaced, and surface markers of macrophages were analyzed by flow cytometry following an additional 48-hour stimulation.

U937-derived M0 macrophages were treated with PF-543 combined with IL-4/IL-13 (Abclonal, Wuhan, China) at a final concentration of 20ng/ml. Following another 24 hours of incubation, the supernatant was collected and stored at −80°C, and cells were harvested for flow cytometry to detect surface markers of M2 macrophages.

### 2.8 Western blot

+PF-543 groups: PF-543 was added to the culture medium of HCT116, SW480, Caco-2, and M2-like macrophages at a final concentration of 20 ng/mL for 24 hours.

NC groups: The same amount of DMSO (the solvent for PF-543) was added to culture medium of HCT116, SW480 and Caco-2, and M2-like macrophages for 24 hours.

Total protein was extracted using RIPA buffer (Sigma Aldrich, Cambridge, MA, USA) supplemented with protease and phosphatase inhibitors (Sigma Aldrich, Cambridge, MA, USA). Protein concentrations were determined using the BCA Protein Assay Kit (PC0020, Solarbio, China). Proteins were separated on 10% SDS-PAGE gels (NCM Biotech, China) and transferred onto PVDF membranes (Millipore, USA). The membranes were incubated with primary antibodies overnight at 4°C, followed by incubation with secondary antibodies for 1 hour at room temperature. Protein bands were visualized using an ECL detection kit (Millipore, USA). The list of antibodies are as follow:

Primary antibodies: GAPDH Monoclonal antibody (Mouse/IgG2b, 1:50000, Proteintech, China); VEGFA (Rabbit/IgG, 1:2000, Abcam plc, England)

secondary antibody: Goat Anti-Rabbit IgG H&L (HRP) (ab97051, 1:5000, Abcam plc, England); Goat Anti-Mouse IgG H&L (HRP) (ab205719, 1:5000, Abcam plc, England).

For quantification, band intensities were analyzed using ImageJ software. The optical density of each target protein band was normalized to the corresponding GAPDH band to account for loading differences. Relative expression levels were calculated and statistically analyzed.

### 2.9 HUVEC tube formation assay

The HUVEC tube formation assay was performed as previously described (3). Matrigel (BD Biosciences, Bedford, MA, USA) was added to 24-well plates and polymerized in a 37°C incubator for 1 hour. HUVECs (1 × 10^5^ cells) were seeded into each well with 400 μl of conditioned medium. Tube formation was observed and photographed under a 10× light microscope at 2, 4, 6, and 8 hours. The total length of tubular structures and the total count of nodes structures were quantified using the Angiogenesis Analyzer plug-in in ImageJ software. Three segments of blood vessel crossing are considered to be a node, and the segment between the two nodes is considered to be the effective blood vessel length.

### 2.10 Flow cytometry

Cells in suspension were collected and fixed with 4% paraformaldehyde for 30 minutes. After fixation, cells were permeabilized using permeabilization buffer (BD Biosciences, Bedford, MA, USA). APC-conjugated anti-CD206 antibody (BD Biosciences, San Jose, CA, USA) was used to label the cells, which were incubated on ice in the dark for 30 minutes. Unstained cells served as negative controls. After thorough washing, single-cell analysis was performed using a FACSCalibur flow cytometer (BD Biosciences, San Jose, CA, USA).

### 2.11 Quantitative real-time polymerase chain reaction analysis

Total RNA was extracted using the Fastagen200 kit (Fastagen, Shanghai, China) and reverse-transcribed into cDNA using the PrimeScript RT Reagent Kit (TaKaRa, Japan). qRT-PCR was conducted using the SYBR Premix Ex Taq™ II kit (TaKaRa, Japan) on a real-time PCR system. Relative mRNA expression levels were calculated using the 2−ΔΔCT method, with GAPDH serving as the internal reference. The primer sequences for CD206 were as follows:

- **Forward**: GGGTTGCTATCACTCTCTATGC
- **Reverse**: TTTCTTGTCTGTTGCCGTAGTT.

### 2.12 Immunohistochemical staining

Six pairs of tumor tissues and adjacent tissues from CRC patients were collected from the Department of General Surgery, First Affiliated Hospital of Xi’an Jiaotong University. Paraffin-embedded sections were deparaffinized, rehydrated, and subjected to antigen retrieval using citrate buffer (Beingmate, China). The sections were blocked with goat serum (Beingmate, China) and incubated overnight with SPHK1 polyclonal antibody (Proteintech, Wuhan, China). Following incubation with mouse secondary antibody (Baiyoutai, China), the sections were stained using diaminobenzidine (DAB) and counterstained. Images were captured using the SlideViewer image analysis system (3DHISTECH). Staining intensity was scored as follows: 0 (negative), 1 (weak), 2 (moderate), and 3 (strong). The percentage of positively stained tumor cells was scored as 0 (0–5%), 1 (6–25%), 2 (26–50%), 3 (51–75%), and 4 (76–100%). The immunoreactivity score (IRS) was calculated by multiplying the staining intensity score by the percentage score. Two independent doctors, blinded to clinical data, assessed the IHC results. IRS > 3 was defined as high expression, while IRS ≤ 3 was classified as low expression. **Supplementary Table S3**: DEGs and pathways associated with sphingolipid metabolism. See **Supplementary Table S3** for details of IRS scores by IHC.

### 2.13 Statistical analyses

Image J was used to detect the protein gray value and the number of angiogenic nodes and connections. Statistical analyses were performed using GraphPad Prism 9.5, SPSS 26.0, and R software. To evaluate data distribution, the Shapiro–Wilk test was applied to assess normality. Levene’s test was conducted to examine homogeneity. For comparisons across two groups where normality was not satisfied (p<0.05), non-parametric Wilcoxon rank-sum test was used, an independent two-sided Student’s t-test was used when both normality and homogeneity assumptions were met. For comparisons across more than two groups, the Kruska-Wallis rank-sum test was used due to violations of the normality assumption. When the Kruska-Wallis test indicated significant group differences (p < 0.05), *post hoc* pairwise comparisons were conducted using Dunn’s test, with p-values adjusted for multiple testing using the Benjamini-Hochberg (BH) method.

For multiple comparisons when there are more than two groups in an experiment, multiple testing corrections were applied using the BH method (43).

Survival analyses were conducted using the Kaplan-Meier method, and differences between groups were assessed with the log-rank test. P-value < 0.05 was considered statistically significant.

## 3 Results

### 3.1 Sphingolipid metabolic landscape in CRC tumor microenvironment at single-cell resolution

**Figure 2:**
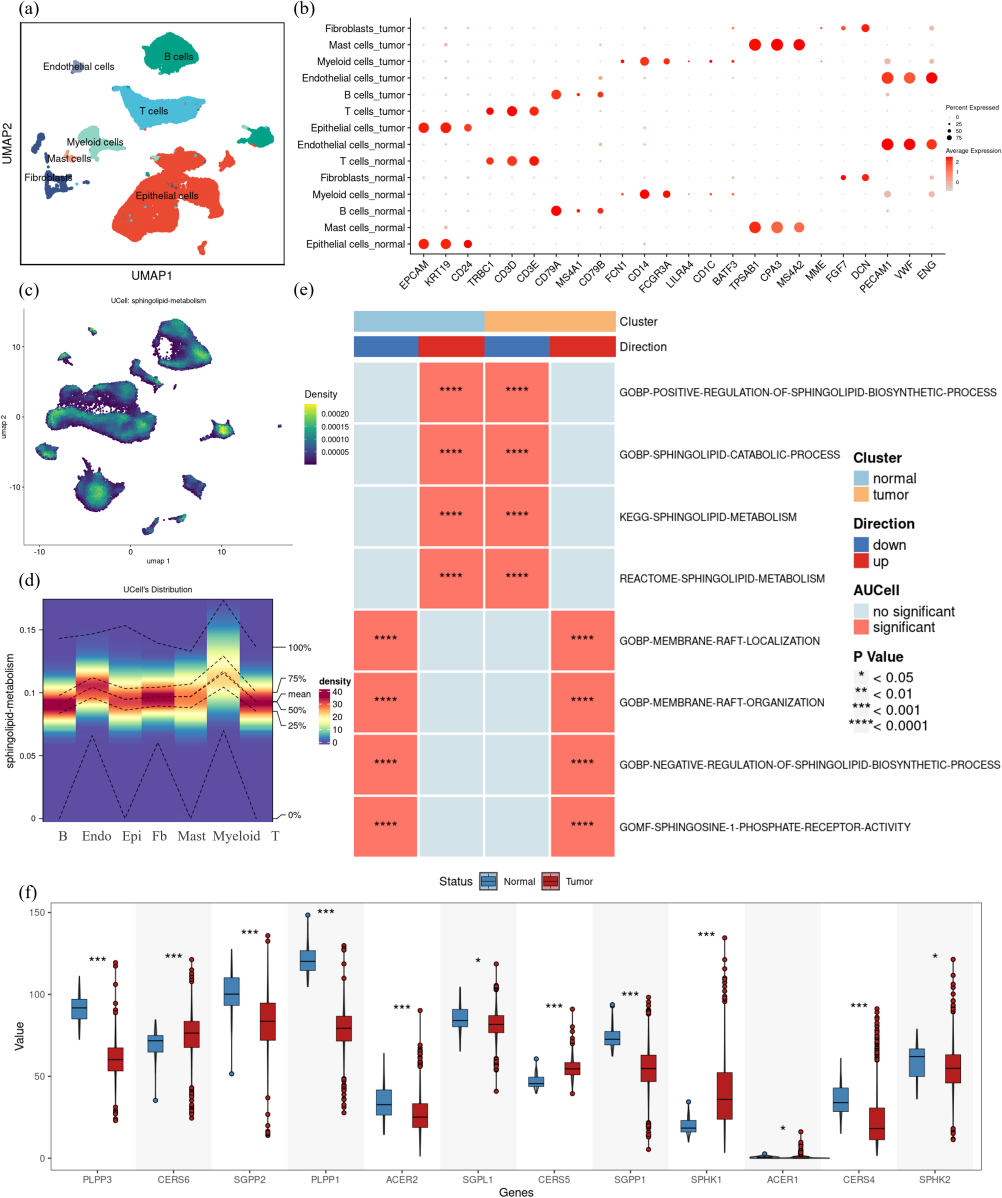
Complexity of sphingolipid metabolic profile in colorectal cancer. **(a)** Single-cell RNA sequencing data from colorectal cancer samples (GSE166555) were analyzed, identifying seven major cell subsets through UMAP clustering: epithelial cells, endothelial cells, fibroblasts, mast cells, myeloid cells, T cells, and B cells. **(b)** Marker genes for the seven cell subsets were visualized, confirming their identities. **(c)** Using the UCell method in the “irGSEA” R package, the sphingolipid metabolic activity scores in CRC tumor tissues were calculated. Regions with a score higher than 0.00015 were considered to exhibit relatively high expression, as shown in the heat map. **(d)** Heatmap visualization of UCell scores across the seven identified cell subsets in CRC tumor tissues indicates that myeloid cells and endothelial cells have significantly elevated sphingolipid metabolic activity. **(e)**AUCell scores comparing sphingolipid metabolism-related pathway activity between normal and tumor tissues demonstrate significant differences. Up-regulated pathways are shown in red, and down-regulated pathways in blue, with asterisks denoting statistically significant differences. **(f)** Expression levels of key enzymes in sphingolipid metabolic pathways were analyzed using TCGA data. Tumor tissues (red) showed significantly higher SPHK1 expression and lower expression of S1P hydrolases (e.g., SGPP1, SGPP2) compared to normal tissues (blue). The differences were evaluated using non-parametric Wilcoxon rank-sum test Multiple testing corrections were applied using the BH method, and statistical significance was denoted as follows: ***p < 0.0001; *p < 0.01; NS, p > 0.01.

By applying dimensionality reduction and batch effect correction on the GSE166555 dataset sourced from the GEO database, we integrated single-cell data derived from 13 CRC tumor tissues and 13 normal tissues. The cells were grouped into seven major cell subsets: epithelial cells, endothelial cells, fibroblasts, mast cells, myeloid cells, T cells, and B cells (**Figure 2a**). Marker gene analysis confirmed the identity of these subsets (**Figure 2b**). Scoring of sphingolipid metabolic activity revealed that myeloid cells and endothelial cells exhibited significantly higher metabolic activity compared to other subsets (**Figures 2c, d**). This suggests that sphingolipid metabolism is not only vital in tumor cells but also closely linked to immune and vascular components of the tumor microenvironment. Significant changes in sphingolipid metabolism were revealed by further function analysis comparing normal and tumor tissues. Although overall sphingolipid metabolism appeared suppressed, tumor tissues enhanced S1P receptor pathway activity, as determined by AUCell and multiple scoring algorithms (**Figure 2e**). Specifically, the balance between ceramide and S1P was disrupted in CRC. Gene expression analysis from the TCGA database revealed significant upregulation of SPHK1, a key enzyme involved in S1P synthesis, and downregulation of genes responsible for S1P hydrolysis, such as SGPP1 and SGPP2 (**Figure 2f**). These findings indicate that tumor cells exploit the dysregulation of sphingolipid metabolism, particularly the S1P pathway, to promote proliferation, immune evasion, and angiogenesis. Additionally, the active sphingolipid metabolism in myeloid cells and endothelial cells underscores their potential role in tumor immunity and vascular remodeling. This positions the S1P-related pathway as a promising therapeutic target.

### 3.2 High S1P expression links to poor prognosis and altered immune dynamics in CRC

**Figure 3:**
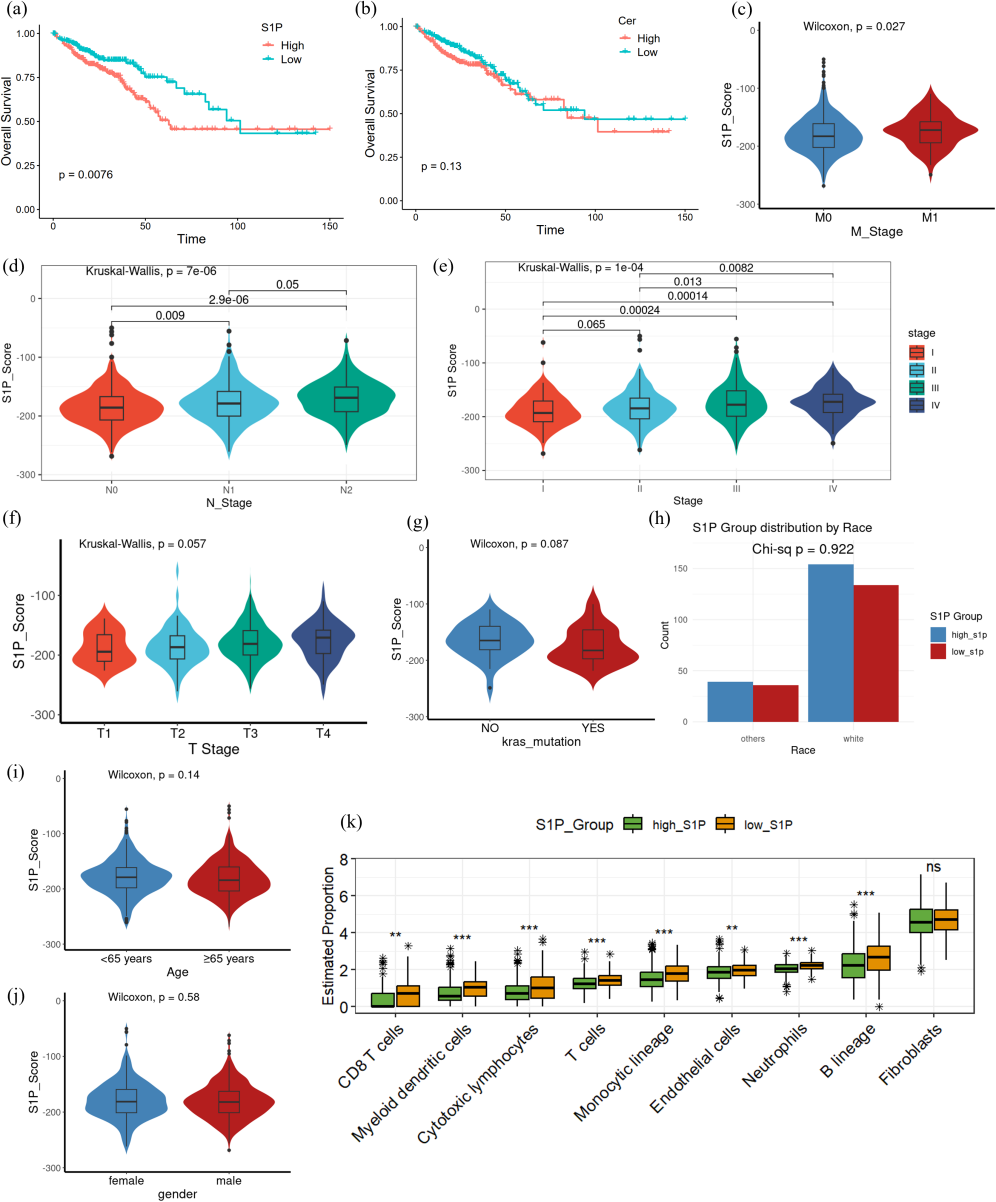
Differences in clinicopathological characteristics and immune infiltration between high and low S1P levels in CRC patients. **(a)** Kaplan-Meier survival analysis comparing overall survival between high and low S1P groups, based on S1P scores calculated from key enzyme differential gene expression; p = 0.0076. **(b)** Kaplan-Meier survival analysis of overall survival between high and low ceramide groups using the same scoring method as in **(a)**; p = 0.13. **(c–g)** Pathological characteristics of CRC patients from the TCGA database, including T stage, N stage, M stage, overall clinical stage and KRAS mutation status. non-parametric Wilcoxon rank-sum test was used to analyze differences between the high and low S1P groups, with p > 0.05 indicating no significant difference. The Kruskal-Wallis (K-W) test was used for multi-group comparisons, with p < 0.05 indicating significant differences. *Post hoc* pairwise comparisons were conducted using Dunn’s test, with p-values adjusted for multiple testing using the Benjamini–Hochberg (BH) method. **(h)** Race of CRC patients from the TCGA database. A Pearson’s chi-square test with Yates’ continuity correction yielded χ² = 0.00954 (df = 1, p = 0.9222). **(i, j)** Clinical characteristics of CRC patients from the TCGA database, including age and gender. **(k)** Immune infiltration analysis using the MCP-counter algorithm. The high S1P group exhibited significantly reduced levels of CD8+ T cells, cytotoxic lymphocytes, neutrophils, and other immune cell subsets compared to the low S1P group. Multiple testing corrections were applied using the BH method, and statistical significance was denoted as follows: ***p < 0.0001; **p < 0.001; NS, p > 0.01.

Building on the observed dysregulation of the S1P pathway in CRC, we further explored its association with clinical features, prognosis, and immune infiltration. To evaluate S1P activity, we calculated S1P scores based on the differential TPM value of key enzymes involved in S1P synthesis and catabolism. After integrating TCGA clinical data for colon and rectal cancer, we assigned each patient an S1P score, reflecting the inferred S1P levels within their tumor microenvironment. Survival analysis showed that the increased synthesis of S1P were significantly associated with poorer overall survival (p = 0.0076; **Figure 3a**). In contrast, the increased synthesis of ceramide showed no significant correlation with prognosis (p = 0.13; **Figure 3b**). Further clinical analysis revealed that the increased synthesis of S1P were correlated with earlier distant metastasis (M stage; p = 0.027; **Figure 3c**), higher lymph node invasion (N stage; p = 7e-06; **Figure 3d**) and advanced clinical stage (p = 1e-04; **Figure 3e**). However, the increased synthesis of S1P were not significantly associated with T stage (p = 0.057; **Figure 3f**), KRAS mutation status (p = 0.087; **Figure 3g**), race (df = 1; p = 0.922; **Figure 3h**), age (p = 0.14; **Figure 3i**) and gender (p = 0.58; **Figure 3j**). Analysis of immune infiltration using the MCP-counter algorithm demonstrated that CRC patients with the increased synthesis of S1P showed decreased infiltration of various immune cell types, such as CD8+ T cells, myeloid dendritic cells, cytotoxic lymphocytes, and neutrophils, when compared to patients with the reduced synthesis of S1P (**Figure 3k**). Using EPIC algorithm, we analyzed the association of SPHK1, the key S1P producing enzyme, and SGPL1, an S1P hydrolase, with immune cell infiltration. We found that in the TCGA colon and rectal cancer data, the RNA expression of SPHK1 and SGPL1 were positively correlated with the extent of macrophage infiltration in the tumor microenvironment, but not with T cells or B cells. (**Supplementary Figure S1**). These findings suggest that the increased synthesis of S1P may contribute to poor prognosis by suppressing immune infiltration and impairing immune responses within the tumor microenvironment, particularly affecting macrophages.

### 3.3 Single-cell transcriptome analysis reveals the pro-angiogenic function of S1P in tumor cells

**Figure 4:**
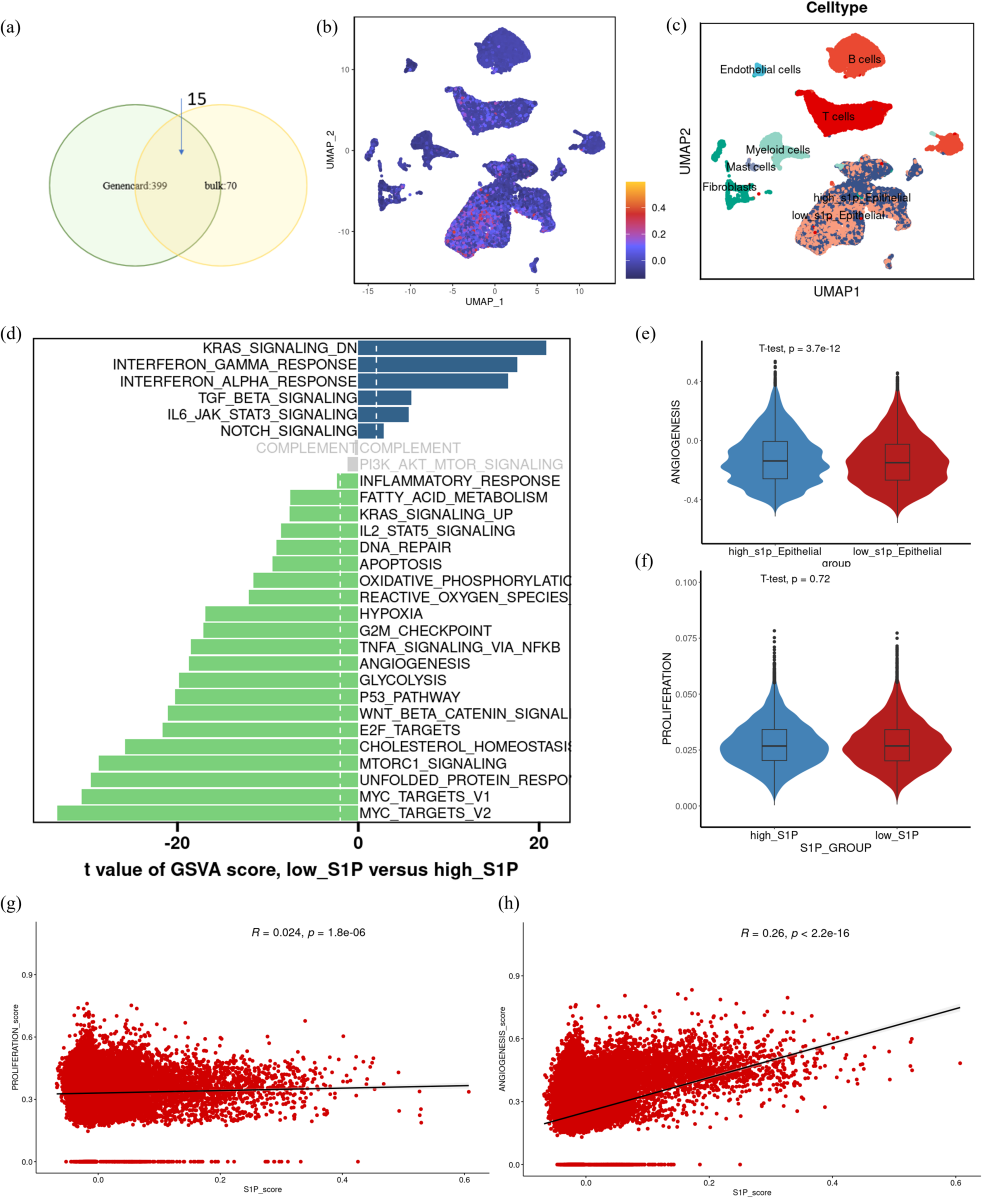
Functional differences in epithelial cells with varying S1P activity at the single-cell transcriptome level. **(a)** Differentially expressed genes between high and low S1P expression groups were intersected with genes having a correlation coefficient > 2 from the GeneCards database to identify a core S1P-related gene set. **(b)** UMAP visualization of S1P activity scores at the single-cell level (scS1P_score), with darker purple indicating lower scores and lower S1P activity. **(c)** UMAP representation of cell subtypes, including high-S1P epithelial cells, low-S1P epithelial cells, T cells, B cells, endothelial cells, fibroblasts, and myeloid cells. **(d)** GSVA functional enrichment analysis of high- and low S1P epithelial cells, showing pathway activity differences. Gray bars indicate no significant enrichment. **(e, f)** Violin plots comparing angiogenesis and proliferation scores between high-S1P and low-S1P epithelial cell subpopulations. Statistical significance was assessed using t-tests. **(g, h)** Correlation analysis showing the relationships between scS1P_score, proliferation score and angiogenesis score.

Based on the observed association between the increased synthesis of S1P and poor prognosis in CRC patients, we conducted single-cell transcriptomic analysis to investigate the specific role of S1P within the TME. We first identified S1P-related genes by intersecting DEGs between high- and low-S1P groups in TCGA datasets with genes strongly correlated with S1P from the GeneCards database (**Figure 4a**). Using this gene set, we quantified S1P activity at the single-cell level and stratified epithelial cells into high and low S1P subpopulations based on the median activity score (**Figures 4b, c**). GSVA enrichment analysis demonstrated that high-S1P epithelial cells exhibited increased enrichment in pathways associated with KRAS signaling, inflammatory response, DNA repair, apoptosis, hypoxia, cell proliferation, and angiogenesis. In contrast, low-S1P epithelial cells were enriched in pathways related to KRAS downregulation, interferon signaling, and TGF-beta signaling (**Figure 4d**). Notably, angiogenesis scores were significantly elevated in the high-S1P epithelial cells compared to the low-S1P epithelial cells (**Figures 4e, f**), suggesting that S1P contributes to the pro-angiogenic phenotype in CRC. Correlation analysis reveals associations between S1P activity score and angiogenesis score, further supporting the pro-angiogenic role of S1P in malignant epithelial cells (**Figures 4g, h**). These findings suggest that S1P facilitates extracellular signal transduction to promote angiogenesis, thereby accelerating CRC progression.

### 3.4 Crosstalk patterns of communication between high-S1P epithelial cells and myeloid cells

**Figure 5:**
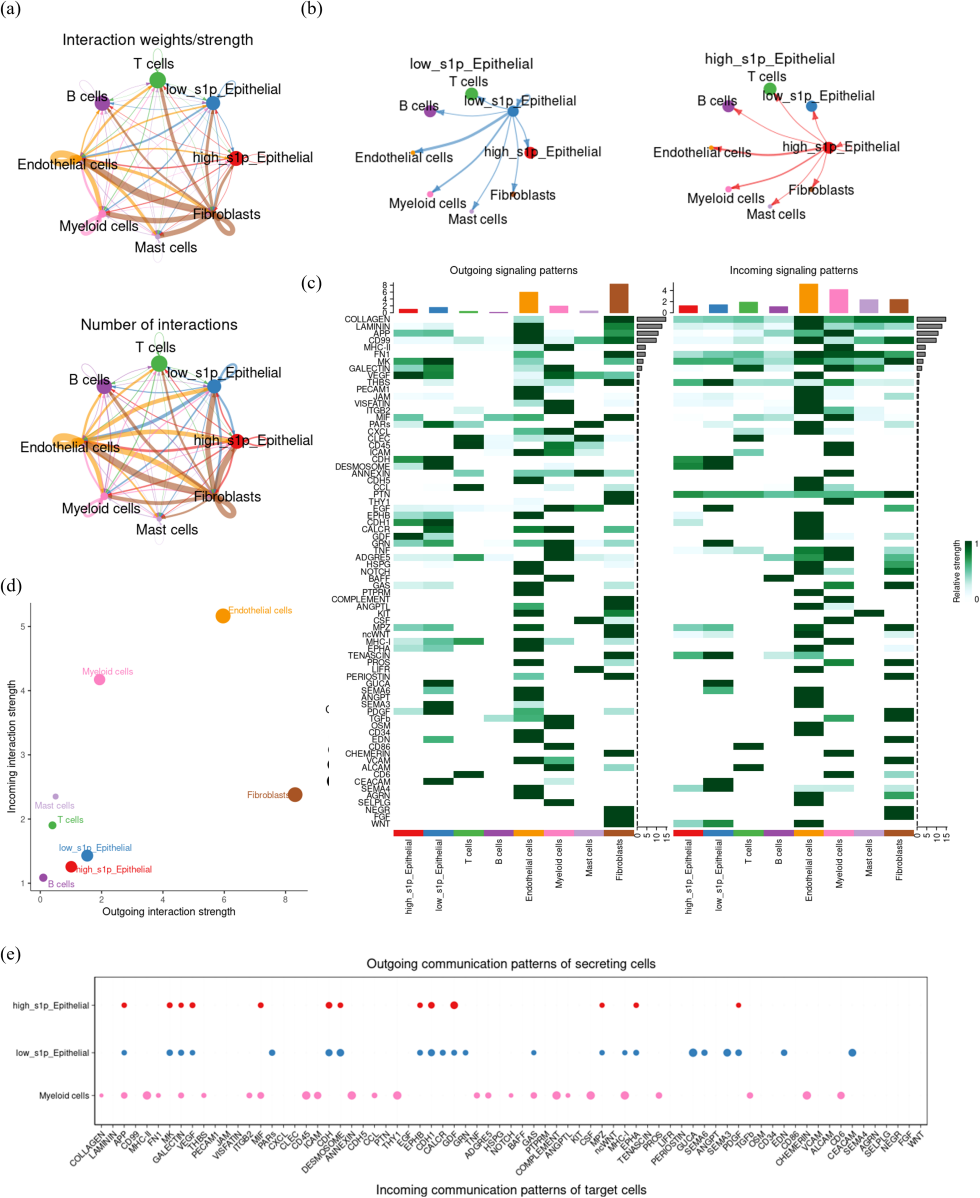
Cell communication crosstalk landscape in CRC tumor microenvironment. **(a)** CellChat network diagram depicting the number and intensity of communications among cell subsets in the CRC tumor microenvironment. Arrows indicate the direction of communication, with thicker and darker lines representing stronger and more frequent communication. **(b)** Comparative visualization of the outgoing signal intensity of high and low S1P epithelial cells. **(c)** Heatmap illustrating the intensity of incoming (input) and outgoing (output) signaling molecules for each cell subpopulation. Darker colors represent stronger communication intensity. **(d)** Dot plot summarizing and visualizing the outgoing (efferent) and incoming (afferent) signal strengths for each cell type, with dot size indicating the number of interactions. **(e)** Detailed characterization of the outward signaling patterns of epithelial cells and the inward signaling patterns of myeloid cells.

To further elucidate the role of S1P in the CRC tumor microenvironment, we employed the “cellchat” package to characterize the crosstalk between various cell types (**Figure 5a**). Initially, our findings indicated that low-S1P epithelial cells presented a stronger communication intensity and a higher number of interactions with other cell types within the TME compared to high-S1P epithelial cells. Nevertheless, this difference in total communication intensity between high- and low-S1P epithelial cells was not significant (**Figure 5b**). Next, we examined the signaling pathways involved in outgoing communication. While no significant differences in the overall output signal intensity were observed between high- and low-S1P epithelial cells, we did observe distinct variations in specific signaling molecules. Notably, high S1P epithelial cells exhibited significantly elevated levels of VEGF, GDF, and MIF signaling pathways, while low-S1P epithelial cells demonstrated higher signaling through MK and MHC-I pathways (**Figure 5c**). Furthermore, combined with the immune infiltration analysis results (**Figure 3k**, **Supplementary Figure S1**), analysis of incoming signaling patterns identified vascular endothelial cells and myeloid cells as the most responsive cell types in the TME, suggesting their potential role in promoting tumor progression through interactions with other cells (**Figure 5d**). This suggests that high-S1P epithelial cells may act on vascular endothelial cells through the VEGF signaling pathway. To further investigate the specific mechanisms of communication between epithelial and myeloid cells, we integrated signals secreted by epithelial cells with those received by myeloid cells. This analysis showed that high-S1P epithelial cells predominantly interacted with myeloid cells through the MIF signaling pathway, whereas low-S1P epithelial cells primarily communicated via GAS and MHC-I pathways (**Figure 5e**). These findings suggest that high S1P expression in epithelial cells enhances MIF signaling, leading to alterations in myeloid cell function and contributing to tumor progression in CRC. By modulating key signaling pathways, epithelial cells in the CRC microenvironment may facilitate the recruitment and reprogramming of myeloid cells, ultimately promoting tumor growth and development.

### 3.5 MIF as a key molecule in the interaction between epithelial cells and myeloid cells

**Figure 6:**
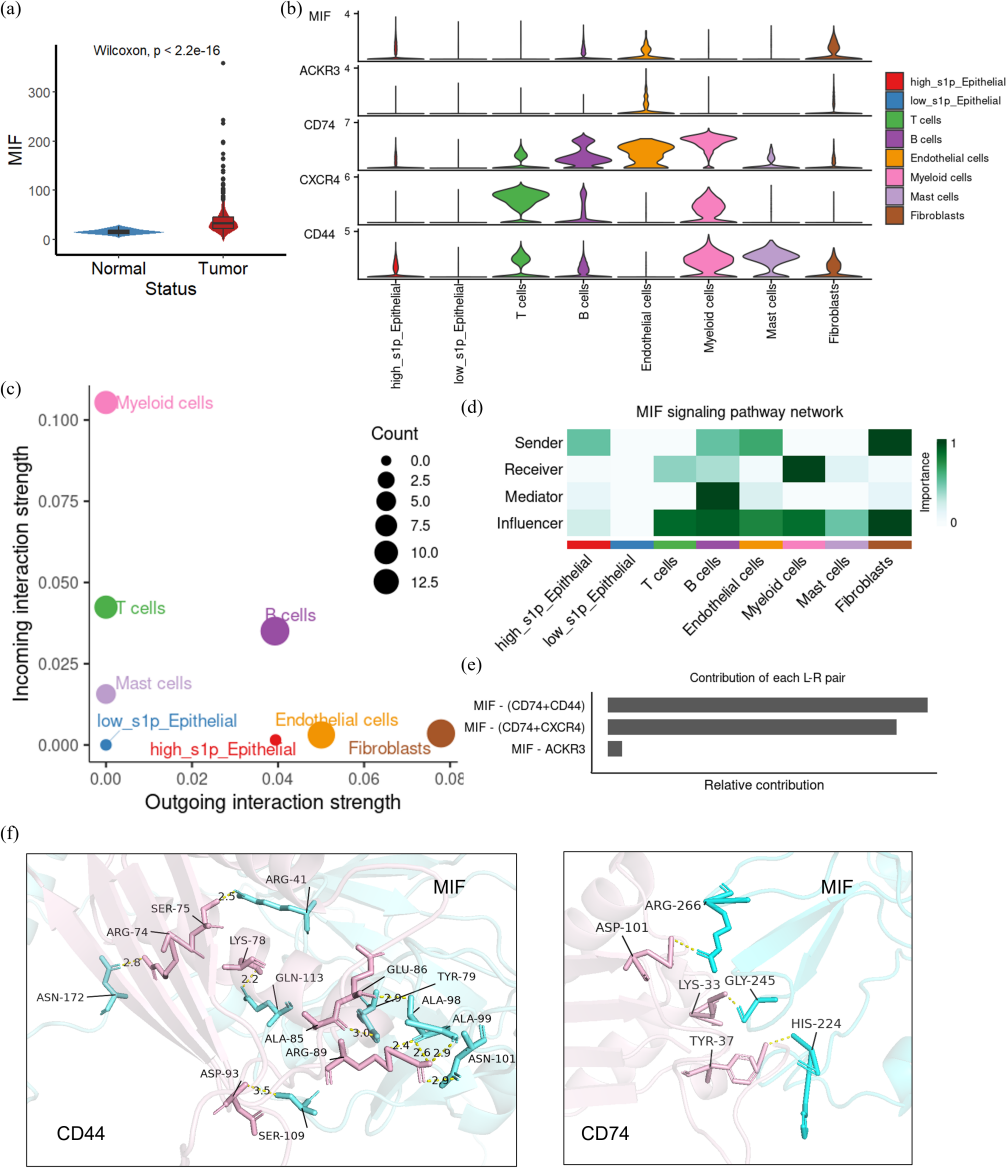
The role of MIF-CD74/CD44 in colorectal cancer (CRC) cell communication. **(a)** Expression of MIF in CRC tissues and normal tissues were compared from TCGA data; **(b)** Violin plot showing the expression of MIF/CD44/CD74 across each cell subset in the single cell dataset; **(c)** Dot plots summarizing the input and output signal intensities in CRC; **(d)** Network centrality analysis identifying the roles of each cell subset in intercellular communication.(senders, receivers, mediators or influencers) **(e)** Quantitative analysis of ligand-receptor pairs involved in MIF-mediated communication; **(f)** Key sites of MIF interaction with CD44/CD74 at the 3D position. In the MIF-CD74 complex, hydrogen bonds were formed between MIF (Ser75, Arg74) and CD74 (Asn172, Lys78). Similarly, in the MIF-CD44 complex, interactions were observed between MIF (Asp101, Arg41) and CD44 (His224, Gly245).

To further investigate the expression and role of MIF in CRC, we compared MIF expression levels between normal and tumor tissues. The results showed that MIF expression was significantly elevated in CRC tumor tissues compared to normal tissues (**Figure 6a**). Subsequently, we visualized the expression of MIF and its interacting ligand-receptor pairs across different cellular subpopulations. High-S1P epithelial cells were identified as the primary source of MIF, while its receptors, such as CD74, CD44, and CXCR4, were predominantly expressed on myeloid cells and T cells (**Figure 6b**). To analyze the role of MIF signaling in the CRC tumor microenvironment, we calculated the input and output signaling intensities of the MIF pathway for each cell subpopulation. Results indicated that the strongest output signals were observed in high-S1P epithelial cells, endothelial cells, and fibroblasts, while myeloid cells demonstrated the strongest input signals (**Figure 6c**). This suggests that high-S1P epithelial cells might influence myeloid cells through the MIF pathway. We further characterized the roles of each cell subpopulation in the MIF signaling network by calculating centrality metrics. High-S1P epithelial cells emerged as the primary senders of MIF signals, while myeloid cells were the primary receivers. Endothelial cells and fibroblasts also played critical regulatory roles as mediators and influencers in the signaling network (**Figure 6d**). Additionally, we calculated the relative contribution of specific ligand-receptor pairs within the MIF signaling pathway. The results showed that the MIF-CD74 and MIF-CD44 pairs contributed significantly to the interaction between epithelial cells and myeloid cells (**Figure 6e**). To explore the molecular basis of these interactions, we employed the AlphaFold3 platform to predict docking sites for the MIF-CD74 and MIF-CD44 complexes and visualized the structures using PyMOL. The structural analysis showed the key hydrogen bond interactions between MIF and its receptors, further reveals the molecular mechanisms by which MIF influences cellular communication in CRC (**Figure 6f**).

### 3.6 A subset of macrophages with high S1P activity has a significant pro-angiogenic effect

**Figure 7:**
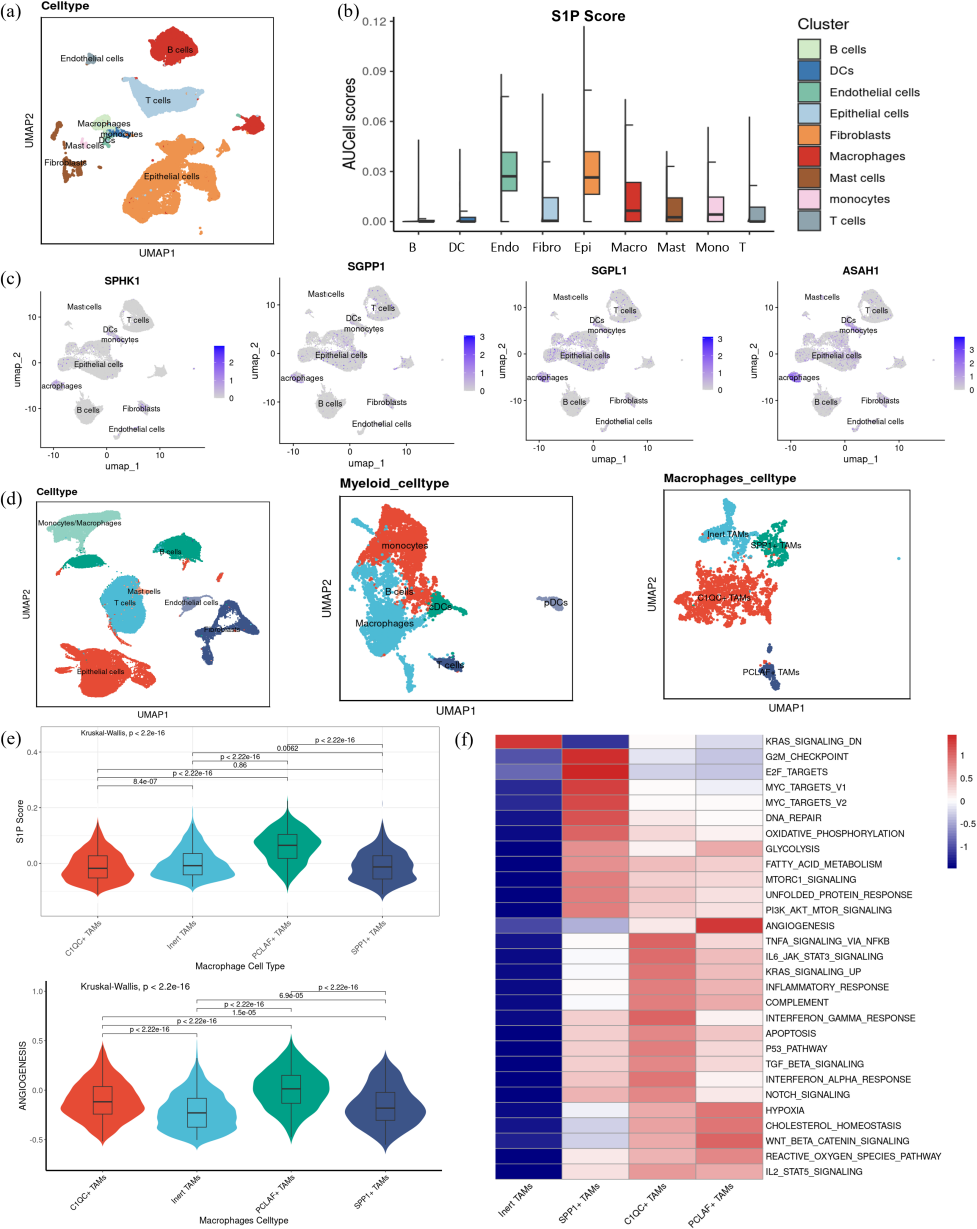
S1P distribution and function in macrophages. **(a)** UMAP visualization displaying the subdivision of myeloid cells into three subsets: dendritic cells, macrophages, and monocytes **(b)** S1P activity was quantified in each cell subset. **(c)** Gene expression levels of key enzymes in the S1P metabolic pathway, including SPHK1, SGPP1, SGPL1, and ASAH1, are visualized across cell subsets. **(d)** UMAP visualization showing dimensionality reduction and clustering annotation after integrating three single-cell datasets, highlighting the classification of macrophages into four subgroups: Inert TAMs, SPP1+ TAMs, C1QC+ TAMs, and PCLAF+ TAMs. **(e)** Violin plots illustrating the S1P and angiogenesis scores across the four macrophage subgroups, with statistical significance determined by Kruskal-Wallis (K-W) analysis for group comparisons and *post hoc* pairwise comparisons were conducted using Dunn’s test, with p-values adjusted for multiple testing using the Benjamini–Hochberg (BH) method. **(f)** Heatmap visualization of KEGG pathway enrichment scores, focusing on hallmark pathways associated with the four macrophage subsets, including angiogenesis, hypoxia, inflammation, and DNA repair.

Building upon the observation that myeloid cells are key recipients of MIF signaling, we sought to identify the specific myeloid cell subsets interacting with high-S1P epithelial cells. To address this, we first characterized the major cell types within the TME, including epithelial cells, endothelial cells, fibroblasts, T cells, B cells, dendritic cells, macrophages, monocytes, and mast cells (**Figure 7a**). Among these, macrophages displayed a significantly elevated S1P activity score compared to other immune cell subsets, highlighting their potential role as primary S1P signal recipients (**Figure 7b**). Next, we examined the RNA expression levels of key enzymes involved in S1P metabolism, including SPHK1, SGPP1, SGPL1, and ASAH1, across different cell types. The results revealed higher metabolic activity in macrophages, reinforcing their potential functional significance in S1P-mediated signaling (**Figure 7c**). This indicates that S1P may directly influence macrophage function, thereby impacting tumor progression. Combined with the previous conclusion that SPHKI and SGPL1 were highly correlated with the level of macrophage infiltration in the transcriptome data (**Supplementary Figure S1**), we focused on macrophages. To further explore the relationship between macrophage S1P activity and angiogenesis, we expanded the single-cell dataset by integrating three datasets comprising 43 tumor samples, focusing on tumor-derived macrophages. These macrophages were classified into four subpopulations based on transcriptional profiles: Inert TAMs, SPP1+ TAMs, C1QC+ TAMs, and PCLAF+ TAMs (**Figure 7d**). Among these subpopulations, PCLAF+ TAMs exhibited the highest S1P activity scores, accompanied by a pronounced pro-angiogenic effect (**Figure 7e**). This suggests that macrophages with elevated S1P activity play a critical role in promoting angiogenesis in the tumor microenvironment. Furthermore, KEGG pathway enrichment analysis demonstrated functional specialization among these macrophage subpopulations. SPP1+ TAMs were enriched in pathways associated with DNA damage and oxidative phosphorylation, C1QC+ TAMs were linked to inflammatory responses, and PCLAF+ TAMs showed significant enrichment in pathways related to angiogenesis and hypoxia (**Figure 7f**). These findings strongly support the notion that macrophages with higher S1P activity are key drivers of angiogenesis, which may contribute to tumor progression by enhancing vascularization.

### 3.7 S1P signaling promotes M2 polarization of macrophages and influences endothelial cells

**Figure 8:**
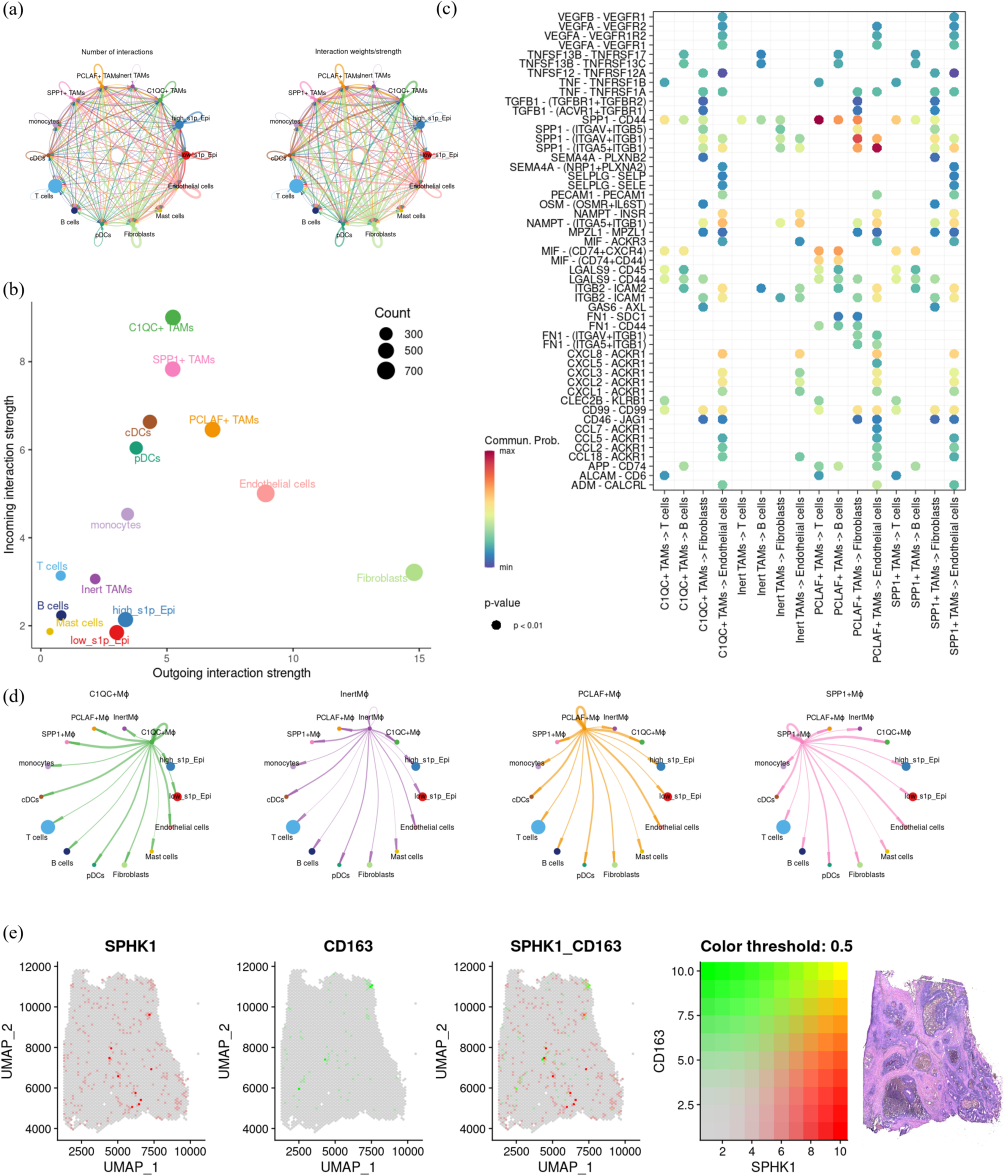
Investigation of cell communication and spatial distribution of macrophage subsets. **(a)** Bubble plot showing receptor-ligand interactions of macrophage subsets with fibroblasts, T cells, B cells, and endothelial cells. **(b)** Dot plot summarizing the afferent and efferent signal intensities of the four macrophage subsets. **(c)** Coil plot representing the aggregated intercellular communication network, calculated using CellChat to determine the number of connections and overall communication probabilities. **(d)** Visualization of intercellular signaling pathways originating from the four macrophage subsets. **(e)** Spatial transcriptomics displaying co-localization of key genes in colon cancer, illustrating the spatial proximity between macrophage subsets and specific gene markers.

To further clarify the role of S1P signaling in macrophage function and intercellular communication, we analyzed the strength and extent of cell-cell interactions using “CellChat” (**Figure 8a**). Among the four macrophage subsets, PCLAF+ TAMs showed the strongest outgoing signals, whereas C1QC+ TAMs showed the strongest incoming signals (**Figure 8b**). Moreover, PCLAF+ TAMs showed closer communication with vascular endothelial cells compared to the other subsets (**Figure 8d**). Further analysis of ligand-receptor interactions revealed that PCLAF+ TAMs primarily acted on T cells and fibroblasts through the SPP1-CD44 signaling axis and influenced vascular endothelial cells via VEGFA-VEGFR2/VEGFR1/VEGFR1R2, but not VEGFB-VEGFR1. Additionally, interactions with vascular endothelial cells were mediated by the SPP1-ITGB1 signaling pathway. (**Figure 8c**). These results suggest that macrophages with elevated S1P levels promote angiogenesis through these specific molecular interactions. It is well established that macrophages can be classified into M1 and M2 subtypes, with M2 macrophages commonly associated with tumor promotion and pro-angiogenic effects. To investigate this further, we used 10X spatial transcriptomics to map the spatial distribution of SPHK1, a key enzyme in S1P synthesis, and M2 macrophages. Notably, these analyses revealed a strong spatial association between SPHK1 expression and M2 macrophages (**Figure 8e**). In conclusion, S1P appears to promote M2 polarization of macrophages, which subsequently enhances angiogenesis by acting on vascular endothelial cells.

### 3.8 Effect of the SPHK1/S1P pathway on tumor cells and macrophages

**Figure 9:**
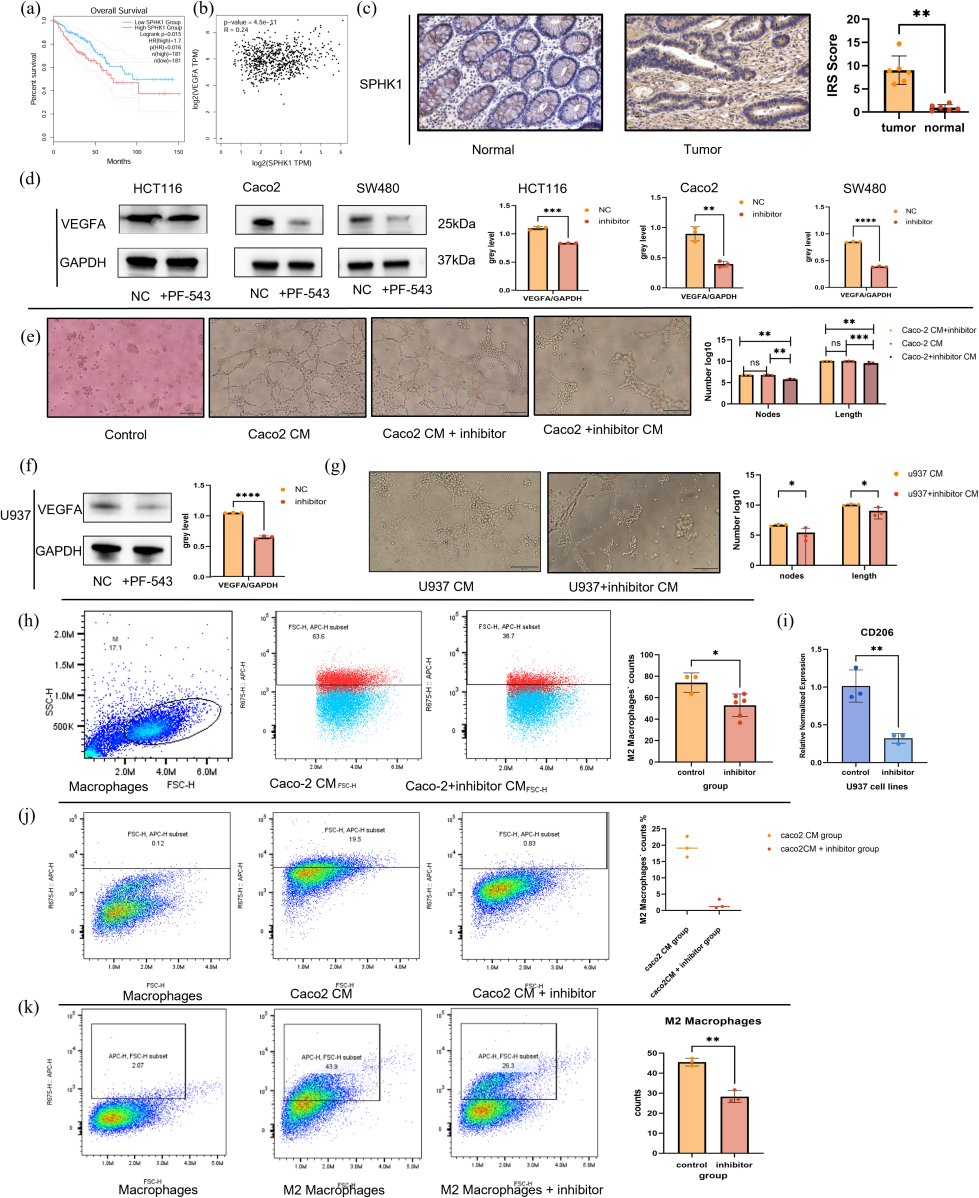
*In vitro* exploration of SPHK1/S1P pathway effects on tumor cells and macrophages. **(a)** Kaplan-Meier survival curves stratified by SPHK1 expression. **(b)** Correlation analysis between SPHK1 and VEGFA expression levels. **(c)** Immunohistochemical staining of SPHK1 in colorectal cancer tumor tissues and adjacent normal tissues (20× magnification). **(d)** Western blot analysis shows VEGFA protein expression in HCT116, Caco2, and SW480 cell lines treated with or without the SPHK1 inhibitor PF-543 for 24h, with corresponding quantification. **(e)** HUVEC tube formation assay. From left to right, the groups are as follows: control group (serum-free medium), Caco2-conditioned medium (Caco2 CM) group, Caco2 CM + inhibitor group, and Caco2 + inhibitor CM group (supernatant collected from Caco2 cells treated with the inhibitor, see Methods for details). After 8 hours of treatment with the respective conditioned media, tube formation was observed and quantitatively analyzed (10× magnification, bar=100μm). The vertical axis represents the count of nodes and the total length of segments(μm), presenting on a logarithmic scale. **(f)** Western blot analysis shows 776 VEGFA protein expression in M2-like macrophages treated ±PF-543. **(g)** HUVEC tube formation assay. From left to right, the groups are as follows: U937 CM group, U937+ inhibitor CM group. The observation time and statistical methods are the same as those in figure 9d (10× magnification, bar=100μ m). **(h)** Flow cytometry analysis of CD206+ M2 macrophages following treatment with Caco2-conditioned medium (Caco2 CM) or inhibitor-treated Caco2-conditioned medium (Caco2 + inhibitor CM) for 48 hours after PMA-induced differentiation of U937 cells into M0 macrophages. Representative flow cytometry plots and statistical quantification are shown from left to right. **(i)** Relative CD206 mRNA expression in macrophages following the same treatments. **(j)** Flow cytometry analysis of M0 macrophage polarization induced by Caco2 CM, with or without PF-543 treatment. **(k)** Flow cytometry analysis of macrophage polarization induced by IL-4 and IL-13, with or without PF-543 treatment. ****p < 0.00001; ***p < 0.0001; **p < 0.001; *p < 0.01.

Previous single-cell analysis demonstrated that S1P not only promotes angiogenesis but also enhances M2 polarization of macrophages. Survival analysis of key enzymes in the S1P axis suggests that patients with higher SPHK1 expression exhibited poorer overall survival (**Supplementary Figures S2**, **S3**, **9a**). Correlation analysis also confirmed that SPHK1 is the key enzyme in the S1P axis (**Supplementary Figures S3**, **9b**). To further investigate these findings, we conducted a series of *in vitro* experiments. First, immunohistochemical staining of SPHK1 was performed on six paired samples of tissues from tumor core area and adjacent normal area in CRC patients. SPHK1 expression was significantly elevated in tumor tissues compared to normal adjacent tissues (**Figure 9c**). Then we used PF-543, a competitive inhibitor that binds to SPHK1, blocking its ability to phosphorylate sphingosine and thereby reducing S1P levels. Western blot analysis revealed a significant reduction in VEGFA protein expression across three CRC cell lines (HCT116, Caco2, and SW480) treated with PF-543 (**Figure 9d**). Tube formation assays revealed that endothelial cells formed tube structures when cultured in Caco2-conditioned medium (Caco2 CM group in **Figure 9e**). However, inhibition of the SPHK1/S1P axis in Caco2 cells with PF-543 significantly suppressed endothelial tube formation (Caco2 + inhibitor CM group in **Figure 9e**). Additionally, when PF-543 was directly added to Caco2 CM, tube structures remain intact (Caco2 CM + inhibitor group). These results indicate that suppression of the SPHK1/S1P axis in tumor cells indirectly inhibits angiogenesis, thereby hindering tumor progression (**Figure 9e**). Furthermore, we examined the role of M2 macrophages in endothelial tube formation. U937 cells were first differentiated into M0 macrophages using PMA, followed by M2 polarization induction with IL-4 and IL-13. Pre-differentiated M2 macrophages were then treated with PF-543. The results showed that PF-543 not only inhibited VEGFA protein expression in macrophages but also suppressed their pro-angiogenic function (**Figures 9f, g**). These findings suggest that the SPHK1/S1P axis influences endothelial tube formation by modulating VEGFA levels in both tumor cells and M2 macrophages, which is in accordance with our bioinformatics analysis.

To further evaluate the impact of PF-543 on macrophage polarization, we treated M0 macrophages with Caco2+inhibitor CM or Caco2 CM. After 48 hours, flow cytometry analysis revealed a significant reduction in CD206+ M2 macrophages in the inhibitor-treated group (**Figure 9h**). At the RNA level, CD206 expression was significantly downregulated (**Figure 9i**). These findings indicate that inhibition of the SPHK1/S1P axis in Caco2 cells indirectly suppresses macrophage M2 polarization. Finally, we assessed the direct effect of SPHK1/S1P on macrophage polarization using flow cytometry. The addition of PF-543 can directly inhibit macrophage polarization, regardless of whether M2 macrophage differentiation was induced by Caco2 CM or IL-4 and IL-13 cytokines. This suggests that SPHK1/S1P modulates macrophage polarization through both direct and indirect mechanisms (**Figures 9j, k**).

In conclusion, SPHK1/S1P axis is hyperactivated in CRC patients with poor prognosis and promotes angiogenesis by increasing VEGFA expression in both tumor cells and M2 macrophages. Additionally, this pathway facilitates macrophage M2 polarization through both direct and indirect mechanisms. These results highlight the SPHK1/S1P pathway as a promising therapeutic target for anti-angiogenic strategies in CRC treatment.

## 4 Discussion

Sphingolipids are a diverse class of lipids integral to cellular membrane dynamics and signaling (12). Their role in metabolic reprogramming, particularly in the TME, has garnered increasing attention due to advancements in metabolomics. Among these, sphingosine-1-phosphate (S1P) is recognized as a potent tumor-promoting lipid in CRC (13). Previous studies have largely focused on tumor cell-intrinsic effects, such as targeting SPHK1 to inhibit S1P synthesis or enhancing SGPL1 to degrade S1P, with the aim of suppressing tumor proliferation (15). However, there has been limited exploration of the role of S1P in intercellular interactions within the TME, particularly at the single-cell transcriptome level. Our study provides novel insights into the role of SPHK1/S1P pathway in macrophages polarization and their crosstalk with tumor cells.

We observed an upregulation of pathways promoting S1P synthesis in CRC tissues compared with normal tissues. Despite an overall decrease in sphingolipid levels, this metabolic shift suggests a critical role for S1P in tumor progression. Importantly, our analysis stratified TCGA CRC patients into high- and low-S1P groups, revealing that the increased synthesis of S1P correlate with poor prognosis, earlier lymph node metastasis, and distant metastasis. While this scoring model reflects S1P-related gene activity, it does not directly measure active S1P levels due to potenti333al limitations from osmotic gradients or receptor functionality. To address this, we identified genes strongly associated with S1P metabolism and assigned S1P activity scores at the single-cell level, enabling detailed functional characterization of S1P in the TME.

Our findings highlight a novel mechanism by which S1P promotes angiogenesis, not only by directly enhancing VEGFA expression in tumor cells but also through its effects on macrophages. High-S1P epithelial cells communicate with macrophages via the MIF-CD44/CD74 axis, driving M2 macrophage polarization—a phenotype known for its pro-angiogenic and immunosuppressive properties. We identified four macrophage subsets in CRC, whose pro-angiogenic ability was positively correlated with S1P activity scores.

Despite these findings, important questions remain. The specific contribution of MIF in the SPHK1/S1P signaling pathway warrants further exploration. MIF has been known to mediate tumor-macrophage crosstalk in other cancers, such as NSCLC, suggesting a potential role in modulating S1P-driven effects in CRC. Investigating whether SPHK1/S1P and MIF act synergistically to amplify immunosuppressive and angiogenic phenotypes could provide valuable insights and a promising combinatorial therapeutic target.

In summary, our study highlights the crucial role of S1P in promoting angiogenesis and macrophage M2 polarization in colorectal cancer at both the bioinformatics and cellular functional levels. Additionally, we constructed a model of CRC intercellular communication based on scRNA-seq data and identified the MIF-CD44/CD74 axis as the potential key pathway linking S1P activity with macrophage-mediated immunosuppression and angiogenesis. These findings significantly enhance our understanding of TME complexity and intratumoral heterogeneity in CRC, paving the way for novel therapeutic strategies. However, the study still has some limitations.

Our study demonstrated that the SPHK1/S1P axis influences CRC angiogenesis through VEGFA. However, the mechanism by which S1P regulates VEGFA remains unclear. Previous research has shown that S1P can act through both receptor-dependent and receptor-independent pathways (16, 17). In general, S1P modulates vascular permeability and tone via S1P receptors (S1PRs) (16). However, after initially examining the relationship between S1PRs and VEGFA at the transcriptomic level, we observed that S1P may primarily act through a receptor-independent pathway (**Supplementary Figure S3**). Due to the complexity of angiogenesis and the limited transcriptomic data available, we cannot yet draw definitive conclusions. Additionally, we found that the RNA expression of UGT8, a key enzyme in the sphingolipid degradation pathway, is also associated with poor prognosis in CRC. This suggests that S1P may be regulated by other critical steps in sphingolipid metabolism. Further studies, including larger bioinformatics datasets and more extensive *in vitro* and *in vivo* experiments, are needed to better understand this complex biological process. Nonetheless, the SPHK1/S1P pathway holds promise as a key target for disrupting CRC progression and improving patient outcomes.

## Data Availability

The original contributions presented in the study are included in the article/**Supplementary Material**. Further inquiries can be directed to the corresponding author/s.

## Supplementary material

Supplementary Figure 1 The immune cell infiltration of SPHK1 and SGPL1 in TCGA-COAD and TCGA-READ datasets.

Supplementary Figure 2 Summary pathway diagram of sphingolipid metabolism.

Supplementary Figure 3 S1P pathway related genes expression and function. **(a)** Kaplan-Meier survival curves stratified by other key components of the S1P pathway related genes expression. **(b)** Correlation analysis showing relationships between the S1P pathway related genes and VEGFA.

Supplementary Figure 4 S1P_score and single cell S1P_score (scS1P_score).

Supplementary Table 1 Detailed information on the datasets

Supplementary Table 2 DEGs and pathways associated with sphingolipid metabolism

Supplementary Table 3 IRS scores of SPHK1 IHC in CRC.

Supplementary Table 4 Calculation and Formulas of S1P_score and scS1P_score.

Supplementary Table 5 Quantification of Western Blot Grayscale Intensity.

Supplementary Table 6 Gene Sets Related to Tumor-Associated Functions.

Supplementary Table 7 The adjusted p-values tables of **Figure 3k** and **2f**.
