# Supplementary figures and images for: Sphingosine-1-phosphate stimulates colorectal cancer tumor microenvironment angiogenesis and induces macrophage polarization via macrophage migration inhibitory factor
Source: Front Immunol. 2025 Jun 16;16:1564213. doi: 10.3389/fimmu.2025.1564213 (PMC12206886; doi:10.3389/fimmu.2025.1564213)

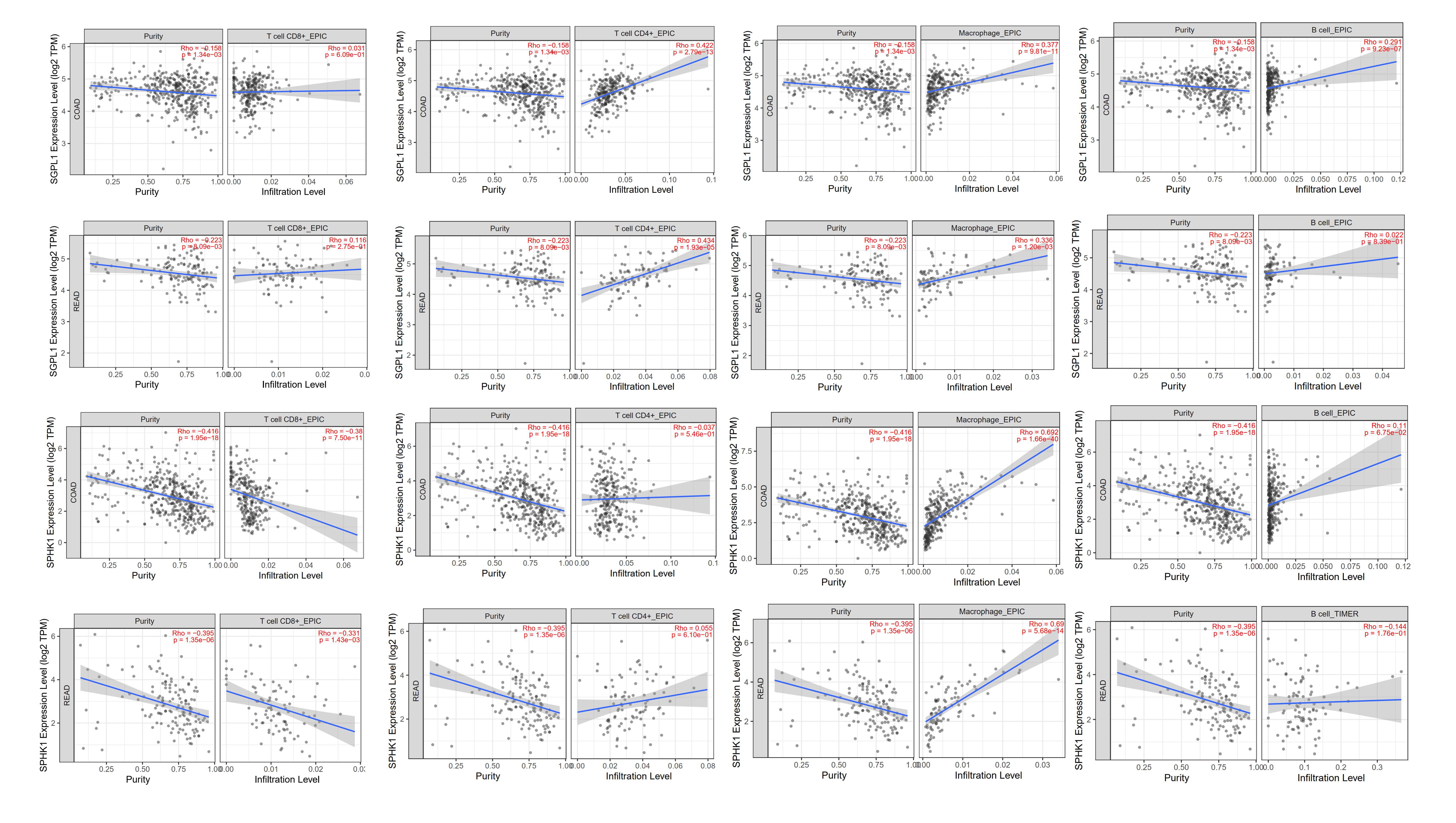

Supplement: Supplementary Figure 1 — The immune cell infiltration of SPHK1 and SGPL1 in TCGA-COAD and TCGA-READ datasets. [file Image1.tif]

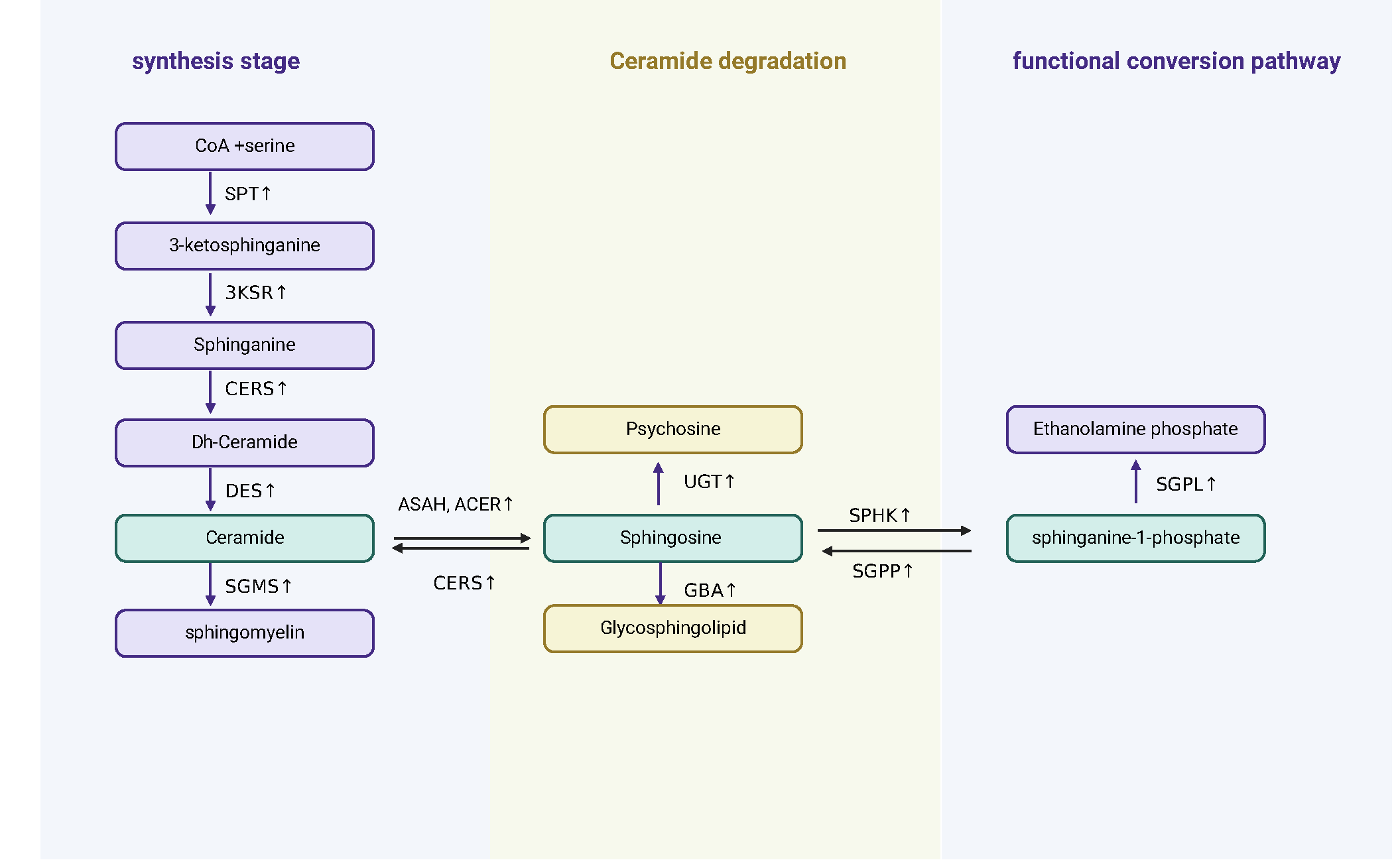

Supplement: Supplementary Figure 2 — Summary pathway diagram of sphingolipid metabolism. [file Image2.tif]

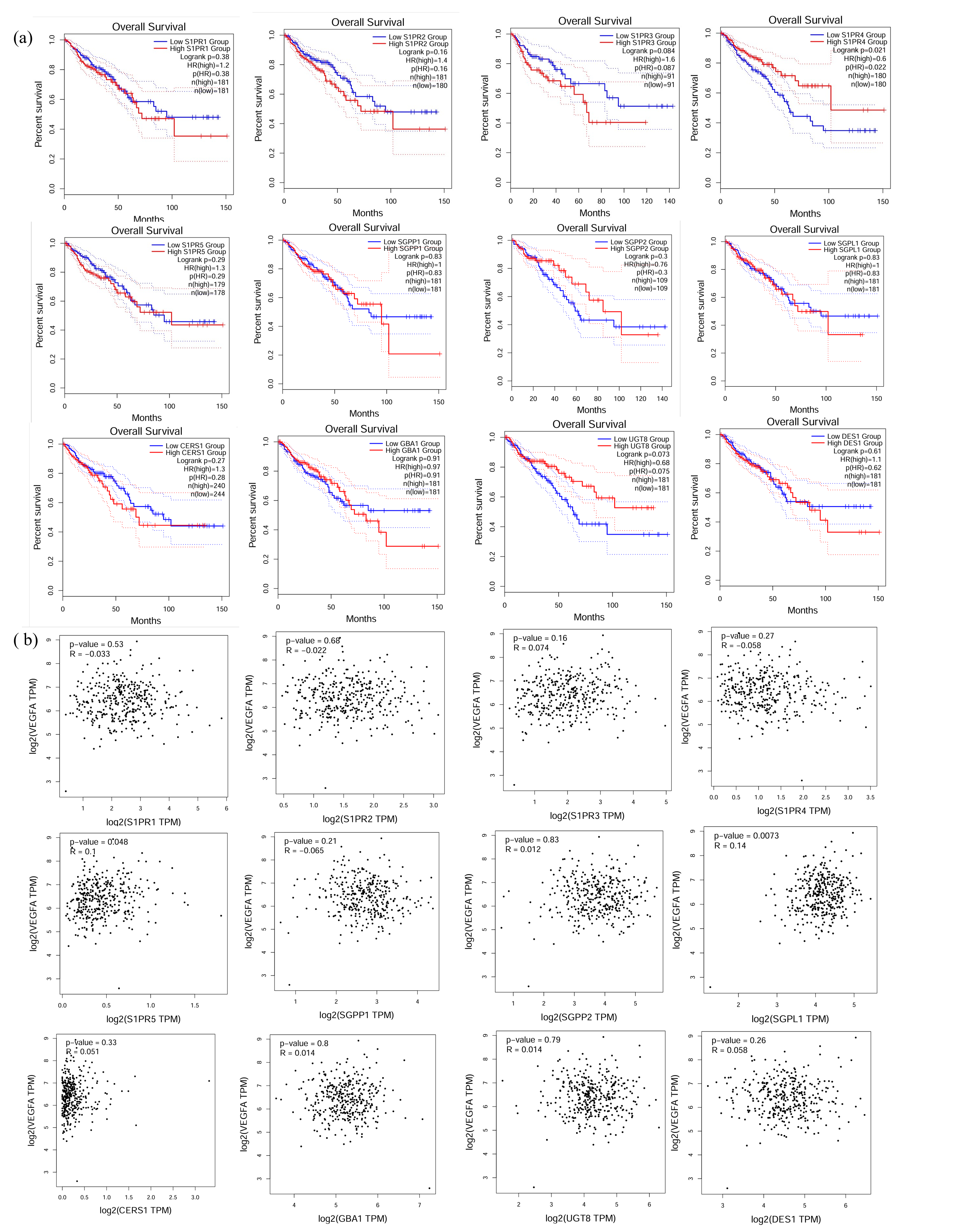

Supplement: Supplementary Figure 3 — S1P pathway related genes expression and function. (a) Kaplan-Meier survival curves stratified by other key components of the S1P pathway related genes expression. (b) Correlation analysis showing relationships between the S1P pathway related genes and VEGFA. [file Image3.tif]

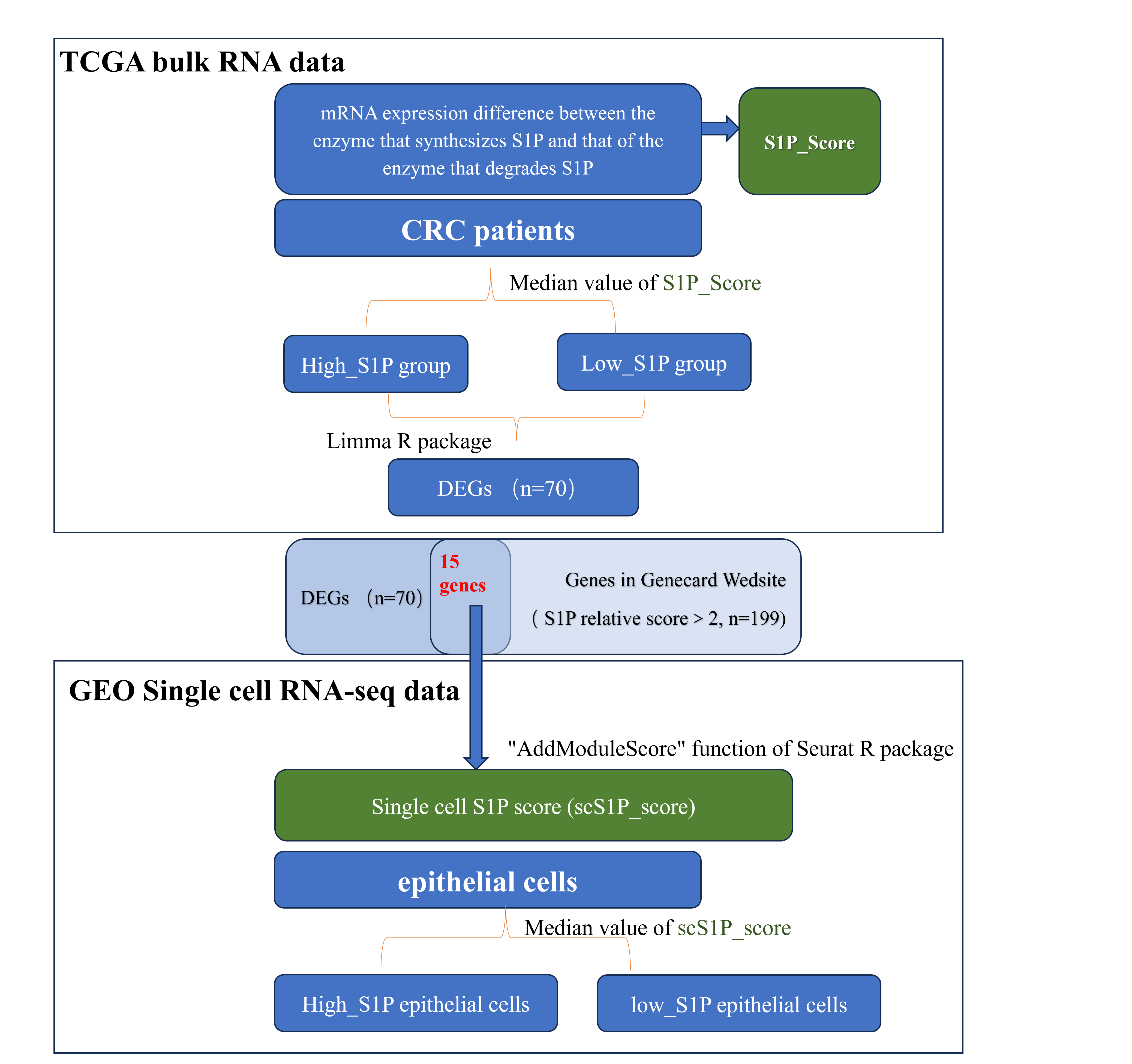

Supplement: Supplementary Figure 4 — S1P_score and single cell S1P_score (scS1P_score). [file Image4.tif]
